# Danhong injection represses diabetic retinopathy and nephropathy advancement in diabetic mice by upregulating microRNA-30d-5p and targeting JAK1

**DOI:** 10.1080/21655979.2021.2006964

**Published:** 2022-03-31

**Authors:** Wei Deng, Dan Huang, HongWu Xie, LiMin Wang, Qun Shen, RongRong Zeng, YuanLian Huang, JianHua Li, Bo Yang

**Affiliations:** a Department of Nephrology, The Fourth People’s Hospital of Chenzhou City, Chenzhou, China; b Department of Ophthalmology, The Fourth People’s Hospital of Chenzhou City, Chenzhou, China; c Department of Endocrinology, The Fourth People’s Hospital of Chenzhou City, Chenzhou, China; d Department of Nephrology, Hengyang Medical School, University of South China, Hengyang, China

**Keywords:** Danhong injection, microRNA-30d-5p, Janus kinase 1, diabetic retinopathy, diabetic nephropathy

## Abstract

Danhong injection (DHI) restrains diabetic retinopathy and nephropathy (DR and DN) advancement in diabetic mice. However, the downstream mechanism of its modulation is not fully studied. Diabetic model mice (db/db mice) were intravenously injected with DHI and corresponding virus particles. MiR-30d-5p and JAK1 were detected. The body weight and fasting blood glucose mice were measured every 4 weeks. The renal tissues and serum of mice were collected, and the contents of creatinine and blood urea nitrogen were biochemically analyzed. IL-6, IFN-γ and TNF-α were detected by ELISA, with the pathological conditions of renal tissues in mice by He staining, and the adjustment conditions by TUNEL. Human retinal pigment epithelium (ARPE-19) cells were selected to induce DR model *in vitro* by high glucose, and exposed to DHI for treatment. The corresponding plasmids were transfected, and miR-30d-5p and JAK1 were detected, with the proliferation ability by plate cloning, apoptosis by flow cytometry, and cell migration ability by Transwell. The angiogenesis ability of cells was assessed by tube formation assay. The targeting relationship between miR-30d-5p and JAK1 was detected. The results manifested that miR-30d-5p was declined in DR and DN, while JAK1 expression was elevated. DHI was able to improve DR and renal injury. DHI could regulate the miR-30d-5p-JAK1 axis *in vivo*, and miR-30d-5p targeted and regulated JAK1. Upregulation of miR-30d-5p or inhibition of JAK1 could improve DR and renal injury. The results implies that DHI can repress the development of DR and DN by elevating miR-30d-5p and targeting JAK1.

## 1. Introduction

Diabetes is a hot health care problem worldwide. In the US, the number of people with diabetes has reached 29 million in 2014 [1]. Recently, the incidence of diabetes in China is elevated versus that in the US and the UK [2]. Diabetes is often accompanied by various complications, such as diabetic nephropathy [3], Cardiac dysfunction [4], retinopathy, Diabetic peripheral neuropathy [5] and so on. Diabetic nephropathy (DN) has been recognized as the most common and serious chronic complication of diabetes for a long time, accounting for about one-third of patients with diabetes [6]. Pathologically, DN is characterized by changes in renal function and structure which can lead to renal insufficiency, renal fibrosis, podocyte damage, and end-stage renal disease [7]. Current treatments for DN contain rosiglitazone [8], tripterygium glycosides [9] and so on. Although there is a certain therapeutic effect in the treatments, it is also accompanied by some adverse reactions, including abnormal liver function, leukopenia, irregular menstruation, etc. Therefore, it is still crucial to explore new treatment strategies for DN.

Diabetic retinopathy (DR), a typical complication of diabetes and a severe eye disease, is the main reason of vision loss and blindness in people with diabetes, affecting their life quality [10]. New evidence suggests that DR is featured by hyperglycemia leading to altered retinal microvascular function and integrity, resulting in progressive retinal ischemia and angiogenesis [11,12]. The treatment approaches of DR consist of laser surgery, intraocular drug injection, vitrectomy, etc., but these conventional treatment methods are difficult to completely cure DR lesions. Therefore, the molecular mechanism of the pathogenesis of DR also requires to be further studied, in order to develop a more novel and effective treatment strategy.

Danhong injection (DHI) is a combination product of Salvianolic Acid and Safflower, with Salvianolic Acid a, Salvianolic Acid C, Hydroxysafflor yellow a as the main ingredients [13]. Studies have shown that DHI can enhance angiogenesis after myocardial infarction [14], alleviate cerebral ischemia reperfusion injury [15]. In addition, DHI has been manifested to repress the progression of DR and DN in diabetic mice [16]. However, the research on the downstream mechanism of its regulation is not enough.

It has been proved that DHI can affect the occurrence of diseases by regulating miRNA expression [17]. In this work, the study of microRNA (miR)-30d-5p was focused on. MiR-30d-5p is a microRNA composed of 2025 nucleotides that play a key part in the remodeling via controlling target genes [18]. It has been reported that miR-30d-5p is crucial in apoptosis during brain development after hypoxic ischemic injury in rats [19]. However, its role in DR and DN is rarely explored.

In this study, it was proposed a hypothesis that DHI participated in regulating DR and DN via miR-30d-5p/JAK1 axis. The study aimed to explore the potential of DHI modulating these pathological behaviors in DR and DN, and the latent molecular mechanisms of DHI functioning in middle and lower reaches of two kinds of complications for offering more impactive gist and strategies for formulating the intervention mechanism of the prevention and treatment of early DN.

## 2. Methods

### 2.1 Experimental animals

Specific pathogen-free (SPF) grade 4-week-old diabetic model (db/db mice) and normal control mice (db/m mice) (Institute of Model Zoology in Nanjing University, China) were fed, given standard food and drinking water, and alternated 12-h light. All experimental animals were carried out with the standards of The Fourth People’s Hospital of Chenzhou City Ethics Committee accordingly.

### 2.2 Grouping

The db/db mice were randomly assigned into the Ctrl (normal saline), DHI, the recombinant adeno-associated virus (rAAV)-miR-negative control (NC), the rAAV-miR-30d-5p, and the rAAV-miR-30d-5p TuDs (DHI) groups with daily intravenous injection in 5 mL/kg weight. Corresponding rAAV (1 × 10^11^ virions) at the age of 8 weeks for mice, and rAAV expressing miR-30d-5p (rAAV-miR-30d-5p) were purchased from Hambio (Shanghai, China) [20].

### 2.3 The sample collection

The body weights of all mice were obtained every 4 weeks. The tail blood of mice was collected every 4 weeks to determine fasting blood glucose (FBG). At the age of 24 weeks, the mice were euthanized via intravenous injection of sodium pentobarbital (45–50 mg/kg i.p), and tissue samples were collected, frozen in liquid nitrogen and stored or fixed with formalin for further researches.

### 2.4 Biochemical analysis

After the animals were euthanized, the blood was collected from the abdominal aorta, coagulated with an anticoagulant, and then centrifuged at 2,000 g. The supernatant plasma was collected for creatinine measurement (C01121; JianCheng Bioengineering Institute, Nanjing, China) and blood urea nitrogen (C01321; JianCheng Bioengineering Institute) with their levels based on commercially available kits.

### 2.5 Enzyme-linked immunosorbent assay (ELISA)

ELISA kit (Luyuan Bode Biotechnology Co., Ltd., Beijing, China) was used. The levels of interleukin-6 (IL-6), interferon-γ (IFN-γ) and tumor necrosis factor-α (TNF-α) in serum of db/db mice were detected. The operation was strictly in accordance with the kit operation instructions.

### 2.6 Assessment of pathological changes in the kidney

The mice kidney tissues were fixed with 4% paraformaldehyde, embedded with paraffin, cut into 4 µm sections for de-affinization, and constructed with hematoxylin staining, hydrochloric acid differentiation, and eosin staining. The obtained sections were dehydrated, cleared with graded ethanol, sealed with neutral adhesive, and then placed under a confocal laser microscope (× 200) to observe the pathological changes [21].

### 2.7 TdT-mediated dUTP-biotin nick end-labeling (TUNEL) staining

In situ cell death detection kit (Roche, Germany) was used to determine apoptosis in the glomerulus using TUNEL method in line with the manufacturer’s protocol. Briefly, kidney tissues were fixed in 4% paraformaldehyde, incubated with a TUNEL reaction mixture, and then treated with an anti-lucifluorescin-POD conjugate. The degree of apoptosis was estimated on the grounds of the average number of TUNEL positive cells per 100 glomerular sections [22].

### 2.8 Cell culture and treatment

Human retinal pigment epithelial cell lines (ARPE-19) (Chinese Academy of Sciences, Shanghai Institutes for Biological Sciences, China) were incubated in a medium of 10% fetal bovine serum (FBS) (Hyclone, Logan, U.S.A.) containing Glutamax [dulbecco’s modified eagle medium (DMEM), Gibco, U.S.A.] and 1% PS (100 units/mL penicillin and 100 mg/mL streptomycin). When treated with d-glucose, ARPE-19 cells were habitually sub-cultured every 3–4 d. After growing to 80% confluence, the cells were seeded into a six-well plate at a rate of 1.5 × 10^4^ cells/well, and treated with d-glucose at different concentrations (0, 5, 10, 20, 30, 50, 100 mM; Sigma-Aldrich, USA) for 24 h. For untreated ARPE-19 cells, 5.5 mM glucose was added to the medium cultured. On the day before cell transfection, ARPE-19 cells were seeded in a six-well plate at a concentration of 2 × 10^4^ cells per well and cultured. After 24 h, the medium was replaced with 0.1 mL DMEM without glucose or FBS and at a range of concentrations treated with or without DHI. Then, the above cells were transfected with miR-30d-5p inhibitor and overexpressed (oe)-JAK1 via 30 μL Lipofectamine 3000 reagent (Invitrogen Life Technologies, USA). The untreated cells were treated as control group (NC). These cells were initially transfected for 6 h and then the medium was replaced with DMEM/ f-12 medium containing 30 mM high glucose (HG). After 24-h incubation, the cells were subjected to the following experiments [23].

### 2.9 Cell viability analysis

Through 3-(4,5-dimethylthiazole-2-yl)-5-(3-carboxyl-methoxyphenyl)-2-(4-sulfonyphenyl)-2 H-tetrazole (MTS, Promega), according to the manufacturer’s agreement, ARPE-19 cells were transfected and cultured under hyperglycemic as described above and then seeded in 96-well plates at a density of 2 × 10^3^ cells/well. Before detection, 100 μL fresh medium was replaced and 20 μL microtubules (MTs) cells were added. The absorbance was measured at 490 nm using Varioskan Flash (Thermo) after 1.5-h incubation with oxygen.

### 2.10 Plate cloning

The 1 × 10^5^ transfected cells and control cells were re-suspended and seeded into 6 cm culture dishes and stored in DMEM containing 10% FBS. Colonies were fixed with methanol, stained with 0.1% crystal violet, and then counted with a light microscope (Olympus, Tokyo, Japan).

### 2.11 Transwell

The cell migration was detected by Transwell migration assay. ARPE-19 cells were pancreatized with 0.25% trypsin, centrifuged, re-suspended and dispersed in serum-free medium. The cell density of the cell suspension was adjusted to 5 × 10^5^ cells /mL. Then, 200 μL cell suspension was added to the upper chamber of the Transwell system (8 μm aperture, Corning, Beijing, China). At the same time, the lower chamber was added with 400 μL 10% FBS medium and incubated. Then the unmigrated cells were removed from the upper chamber, and the cells passing through the membrane were fixed with 4% paraformaldehyde, stained with 0.5% crystal violet, and rinsed with tap water. Finally, the membrane cells were dried and counted under an inverted microscope [24].

### 2.12 Flow cytometry

The experiment was conducted in strict accordance with the instructions of Annexin-V-Fluorescent Iisothiocyanate (FITC)/ Propiroiodide (PI) Apoptosis Detection Kit (Chongqing Huaya Stem Cell Technology Co., Ltd., Chongqing, China). The cells in each group were collected and cultured to a concentration of about 80%, and centrifuged. About 1 × 10^6^ cells were collected and resuspended with 100 μL type 1 buffer, and stained with 5 μL Annexin-VFITC and 5 μL PI (Shanghai Jianglai Biotechnology Co., Ltd., Shanghai, China). The cell apoptosis was detected by flow cytometry.

### 2.13 Tube formation test

The capillary tube forming test was carried out to study the tube forming ability of HREC. The frozen Matrigel (BD Biosciences. Shanghai. China) was thawed overnight in the refrigerator and then added to a pre-cooled 96-well plate. After the gel was solidified, the cells were immediately seeded on the gel at a density of 7 × 10^3^ cells per well. The plate was moistened and incubated. The following parameters are used for quantification. A tube is thought of as a tubular structure that runs from one branch point to another or a loose end. A ring is a closed (or nearly closed) region in a tube that meets the roundness condition. Images of the tubular network were captured under a microscope and quantification of capillaries was assessed using the Image software [25].

### 2.14 Western blot

The right kidney was lysed with a RIPA lysis buffer (Thermo Fisher Scientific, Waltham, MA, USA) containing a mixture of proteases and phosphatase inhibitors (Abcam, Cambridge, MA, USA). The supernatant was centrifuged at 12,000 rpm for collection, and the total protein concentration was determined by bicinchoninic acid (BCA) method. Proteins were separated by electrophoresis and transferred to Polyvinylidene fluoride (PVDF) membranes (Millipore, USA). After being sealed in 5% skimmed milk, the membrane was incubated with anti-JAK1 (1:1000; 3331; Cell Signaling Technology), and GAPDH (1:1000; 2118; Signaling Technology) overnight, and horseradish peroxidase (HRP)-labeled anti-mouse or anti-rabbit secondary antibodies (1:5000; ZSGB-Bio, Beijing, China), and developed with enhanced chemiluminescence (Thermo Fisher Scientific, USA). Images are captured by an image acquisition system (Gel Doc XR+; BioRad, USA), and the gray value is determined by ImageJ software.

### 2.15 RT-qPCR

Total RNA was extracted from tissues or cells using Trizol reagent (Invitrogen, Waltham, MA, USA). The concentration and purity of RNA were measured by nanometer droplet spectrophotometer. As instructed, the complementary DNA (cDNA) was synthesized by reverse transcription of 1 μg total RNA using the PrimeScript RT kit (Promega, Madison, WI, USA). With cDNA as a template, RT-qPCR was performed using SYBR Premix Ex Taq^TM^ kit (Takara, Otsu, Japan). The internal reference of JAK1 is GAPDH, and miR-30d-5p with U6. The primers were synthesized by RiboBio (Guangzhou, China).

**Table:** 

Genes	Forward (5’-3’)	Reverse (5’-3’)
JAK1	CATGGTGGAAGAGTTTGTGGAA	CAGCTGTTTGGCAACTTTGAATT
GAPDH	CGTGGGCCGCCCTAGGCACCA	TTGGCTTAGGGTTCAGGGGGG
MiR-30d-5p	GCCTGTAAACATCCCCGAC	GTGCGTGTCGTGGAGTCG
U6	GCTCGCTTCGGCAGCACA	GAGGTATTCGCACCAGAGGA

### 2.16 Luciferase assay

The wild-type (WT) or mutant type (MUT) 3-untranslated region (UTR) of the JAK1 sequence was constructed and cloned into the pGL3 vector (Promega). HEK293T cells (5 × 10^3^) was seeded into 96-well microplates and cultured. Subsequently, the cells were co-transfected with firefly luciferase reporter vector by Lipofectamine 2000 (Invitrogen), phRL-TK vector (renin luciferase control reporter vector) (Promega), and miR-185/control mimics. The cells were collected and the luciferase activity was detected using a dual luciferase reporting system (Promega Corporation, Madison, WI, USA) [26].

### 2.17 Statistical analysis

Statistical software SPSS 21.0 (SPSS, Inc, Chicago, IL, USA) was applied to analyze the data. Kolmogorov–Smirnov test manifested that the data were normally distributed, and the results were expressed as mean ± standard deviation. The t-test was used for comparison between the two groups. One way analysis of variance (ANOVA) was used for comparison between groups, and Fisher’s least significant difference t test (LSD-t) for pair comparison after ANOVA analysis. Enumeration data were expressed by ratio or percentage, and the chi-square test was applied for comparative analysis. *P* for bilateral test, *P* < 0.05 indicated that the difference is statistically significant.

## 3. Results

In this study, it was speculated that DHI could alleviate DR and DN via elevating miR-30d-5p and repressing JAK1. The exploration of the influence of DHI on DR and DN advancement and its possible downstream molecular mechanism offers a brand-new target for DR and DN’s therapy.

### 3.1 DHI can improve DN

**Figure 1.**
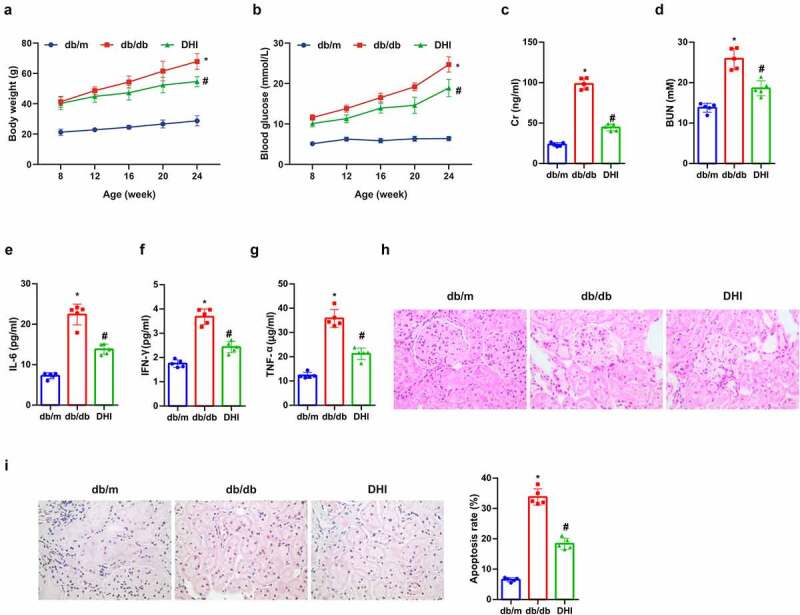
DHI improves DN.

Blood glucose and body weight of the db/db mice were higher versus the control group, while those of the DHI mice were apparently reduced (Figure 1(a,b)). Cr and BUN in serum are well-known indicators of renal function whose abnormal upregulation is related to renal function abnormalities. The blood from the abdominal aorta was collected to explore the effects of DHI on renal function in mice. The results showed that the above indexes in the db/db mice were clearly elevated in contrast with the db/m mice, while they were distinctly decreased under the influence of DHI. This suggests that DHI can improve renal dysfunction by down-regulating Cr and BUN in serum in db/db mice (Figure 1(c,d)). Inflammatory cytokines are crucial in the microenvironment of DN lesions, among which IL-6 is involved in the risk of DN, and IFN-γ in delayed immune response in DN, and TNF-α in damage to the glomerular permeability barrier [27]. The effect of DHI on inflammatory response was studied in db/db mice. It was manifested that the concentrations of IL-6, IFN-γ and TNF-α in the serum of db/db mice were obviously elevated versus the db/m mice, while clearly reduced in the intervention groups (Figure 1(e–g)). HE staining and TUNEL analysis were performed on the kidney of db/db mice to observe the pathological changes. The HE staining results suggested that versus the db/m mice, the glomerular hypertrophy, mesangial matrix dilatation and partial atrophy of renal tubules in the db/db mice were apparently improved under the influence of DHI. It was revealed in TUNEL results that the proportion of positive apoptotic cells in the db/db mice was clearly up-regulated versus the db/m mice, and the high apoptotic level was manifest reduced under the intervention of DHI. All the above results suggested that DHI has different degree and different aspects of renal protection in db/db mice (Figure 1(h,i)).

(a) Blood glucose test every 4 weeks; (b) weight check every 4 weeks; (c) creatinine measurement at 24 weeks of age; (d) urea nitrogen detected at 24 weeks of age; (e-g) inflammatory markers IL-6, IFN-γ and TNF-α contents in serum of db/db mice; (h) the pathological condition of renal tissues detected via HE staining; (i) the renal tissue apoptosis detected via TUNEL staining. The data were expressed as mean ± SEM, (a&b) n = 10; C-I, n = 5; * *P* < 0.05, versus db/m; # *P* < 0.05, versus db/db control.

### 3.2 Overexpression of miR-30d-5p alleviates renal dysfunction in db/db mice

**Figure 2.**
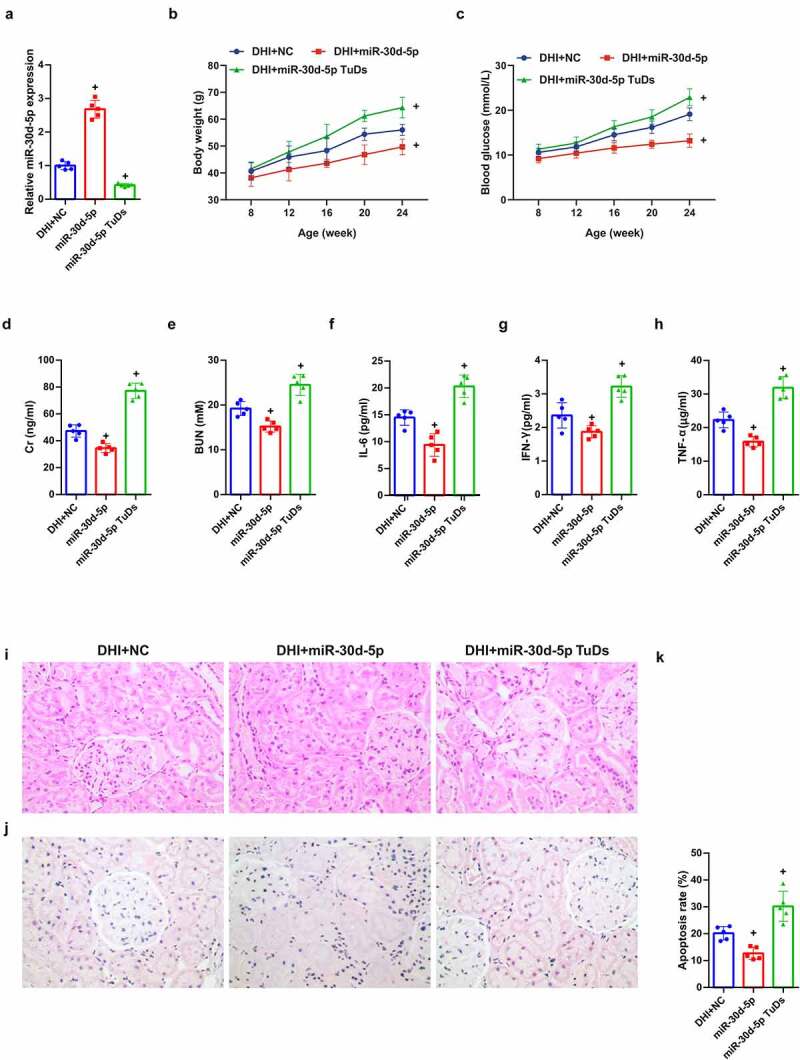
Overexpression of miR-30d-5p alleviates renal dysfunction in db/db mice.

MiR-30d-5p in DN was explored, mature miR-30d-5p in mice was manipulated via rAAV system, manifesting that miR-30d-5p was elevated via rAAV-miR-30d-5p, but reduced via rAAV-miR-30d-5p TuDs (Figure 2(a)). Additionally, blood glucose and body weight were up-lifted in db/db mice introduced with rAAV-miR-30d-5p, while descended after rAAV-miR-30d-5p TuDs treatment (Figure 2(b,c)). Notably, miR-30d-5p overexpression alleviated diabetes-induced renal insufficiency, revealed by a reduction in Cr and BUN in serum (Figure 2(d,e)), whereas miR-30d-5p knockout by rAAV-miR-30d-5p TuDS aggravated the effects (Figure 2(d,e)). In addition, the concentrations of IL-6, IFN-γ and TNF-α in the serum of mice were declined via overexpressed miR-30d-5p, apparently improving the pathological condition and reducing the apoptotic cells (Figure 2(f–j)). All data suggested that miR-30d-5p ameliorates renal injury in diabetic mice.

(a) The relative expression of miR-30d-5p in renal tissues detected via Real-time PCR; (b) blood glucose test every 4 weeks; (c) weight check every 4 weeks; (d) creatinine measurement at 24 weeks of age; (e) urea nitrogen detected at 24 weeks of age; (f-h) inflammatory markers IL-6, IFN-γ and TNF-α contents in serum of db/db mice; (i) the pathological condition of renal tissues detected via HE staining; (j) the renal tissue apoptosis detected via TUNEL staining. The data were expressed as mean ± SEM, (b&c) n = 10; (a&d–j), n = 5; + *P* < 0.05, versus DHI + NC.

### 3.3 DHI can regulate the miR-30d-5P-JAK1 axis *in vivo*

**Figure 3.**
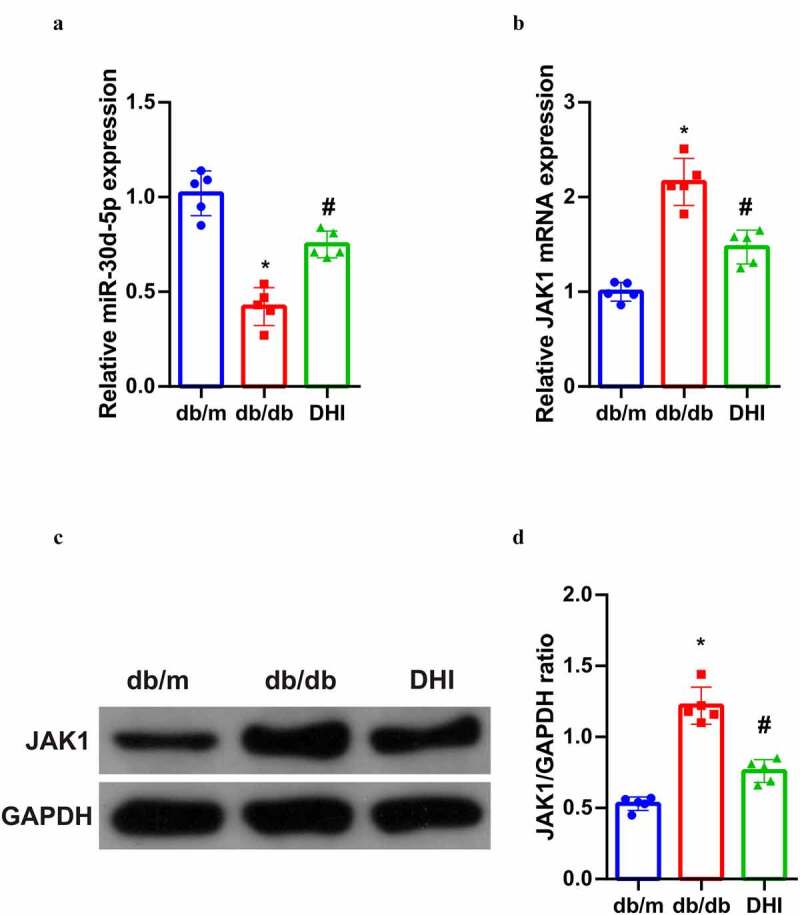
DHI regulates the miR-30d-5p-JAK1 axis *in vivo.*

In order to further understand the potential mechanism of DHI’s anti-DN effect, its relationship with miR-30D-5P-JAK1 axis was analyzed, suggesting that miR-30d-5p was abnormally down-regulated and JAK1 was abnormally up-regulated (transcription and protein levels) in the kidney tissues of db/db mice, and this abnormal imbalance was obviously alleviated by DHI interference. (Figure 3(a–d))

(a/b) The expression of miR-30d-5p and JAK1 in renal tissues detected by qPCR; (c/d) the protein expression of JAK1 in renal tissues detected via Western Blot. The data were expressed as mean ± SEM, n = 5; * *P* < 0.05, versus db/m; # *P* < 0.05, versus db/db control.

### 3.4 DHI treatment can alleviate *in vitro* cell damage caused by DR

**Figure 4.**
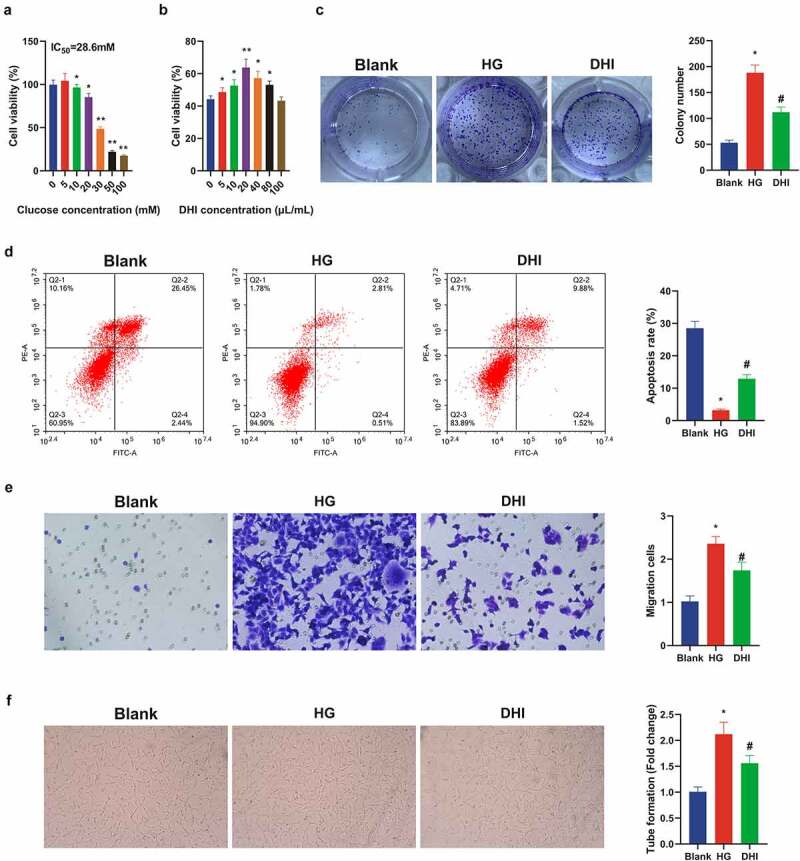
DHI treatment mitigates *in vitro* cell damage in DR.

A literature has revealed that DR is also one of the complications of diabetes mellitus (DM) [28], and DR model was induced via HG in ARPE-19 cells. For detection of DHI on DR, ARPE-19 cells were first treated with HG. The MTT test results in Figure 4(a) initially manifested that ARPE-19 cell viability was distinctly inhibited after introduced with different concentrations of d-glucose. The IC50 value of glucose was calculated as 28.60, and 30 mM glucose was selected as HG to induce the damage of ARPE-19 cells for further experiments.

(a/b) Cell viability detected by MTT assay; (c) cell proliferation detected by plate cloning; (d) apoptosis detected via Flow cytometry; (e) cell migration detected via Transwell assay; (f) the ability of cytological tubule formation tested via Tubule formation assay. The data were expressed as mean ± SEM, N = 3; * *P* < 0.05, versus Blank; # *P* < 0.05, versus HG.

ARPE-19 cells were then exposed to a range of concentrations of DHI with measurement of the cell viability. The cell viability was apparently elevated via DHI treatment (10–80 μL/mL) versus HG group (Figure 4(b)). Therefore, the optimal dose of DHI was selected as 20 μL/mL for subsequent experiments.

Additionally, the proliferation and apoptosis levels of ARPE-19 cells were detected after being treated with 20 μL/mL DHI. It was found that the proliferation of ARPE-19 cells treated with HG was strengthened, and the apoptosis rate was apparently reduced versus the control group, whereas this phenomenon was obviously improved after DHI treatment (Figure 4(c,d)). Transwell revealed that the migration ability of ARPE-19 cells was clearly decreased after DHI treatment (Figure 4(e)). In order to investigate whether DHI had an influence on angiogenesis, tube formation test was constructed to evaluate the tubular formation of HRECs, indicating that the number of capillary-like structures was clearly reduced in the DHI group in contrast with the HG group (Figure 4(f)). These experiments manifested that DHI treatment could alleviate *in vitro* cell damage in DR.

### 3.5 DHI upregulates miR-30d-5p and targets JAK1 *in vitro*

**Figure 5.**
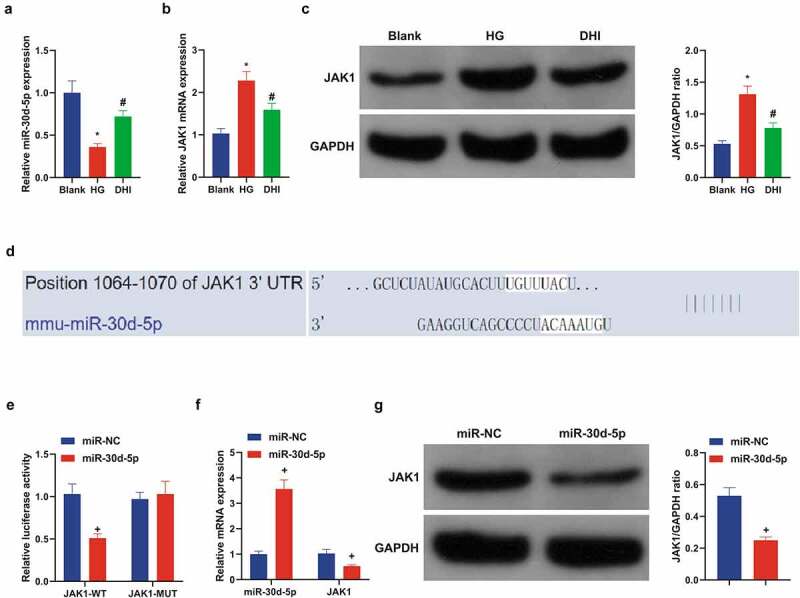
DHI upregulated miR-30d-5p and targeting JAK1 *in vitro.*

It has been shown in previous experiments that DHI regulates the miR-30d-5p-JAK1 axis in mouse kidney tissues. We hypothesized that this phenomenon was similar in an *in vitro* model of HG-induced ARPE-19, so the mechanism of its action was further explored in an *in vitro* model. Analysis suggested that miR-30d-5p was abnormally curbed and JAK1 was abnormally up-regulated in the cells, which was clearly alleviated after DHI (Figure 5(a–c)). Further, the mechanism between miR-30d-5p and JAK1 was specifically studied. Firstly, the prediction site of miR-30d-5p and JAK1 was discovered through the bioinformatics website (Figure 5(d)). Subsequently, through the dual luciferase reporting assay, miR-30d-5p only reduced the level of JAK1-WT. However, there was no obvious effect on JAK1-MUT (Figure 5(e)). JAK1 expression was detected after miR-30d-5p was upregulated in the cells, and JAK1 was apparently decreased (Figure 5(f,g)). The above experiments once again proved that DHI can regulate the miR-30d-5p-JAK1 axis, and miR-30d-5p can target and regulate JAK1.

(a/b) The expression of miR-30d-5p and JAK1 in ARPE-19 cells detected by qPCR; (c) the protein expression of JAK1 in ARPE-19 cells detected via Western Blot; (d) the binding sites of miR-30d-5p and JAK1 predicted via Bioinformatics website (TargetScan); (e/f) the expression of miR-30d-5p and JAK1 after upregulation of miR-30d-5p detected by qPCR. (c) The protein expression of JAK1 after up-regulation of miR-30d-5p detected via Western Blot. The data were expressed as mean ± SEM, N = 3; * *P* < 0.05, versus Blank; # *P* < 0.05, versus HG; + *P* < 0.05, versus miR-NC.

### 3.6 Elevated miR-30d-5p or depressive JAK1 can restrain the development of DR model *in vitro*, further enhancing the protective effect of DHI *in vitro*

**Figure 6.**
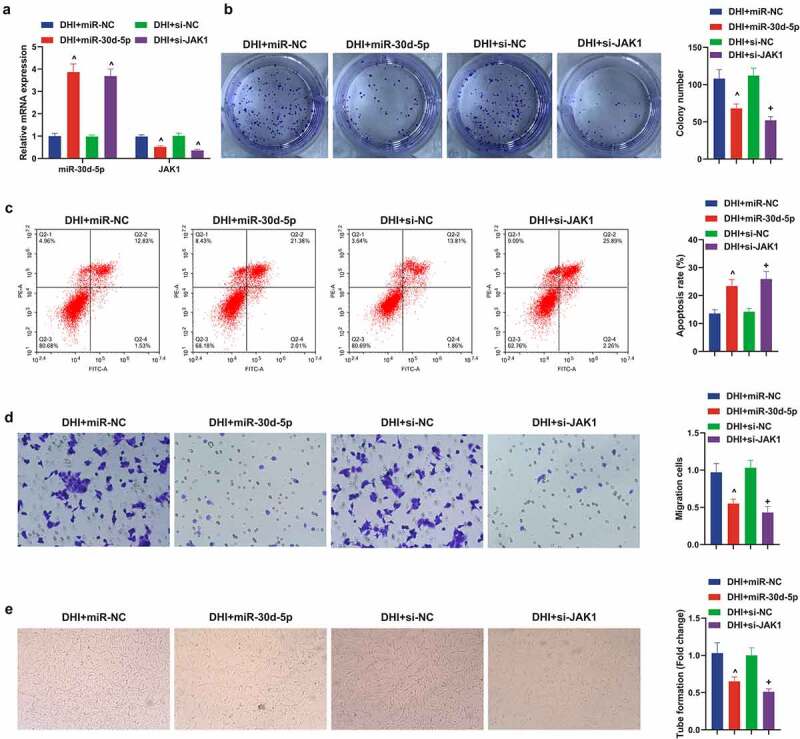
Down-regulating miR-30d-5p or up-regulating JAK1 can facilitate the development of DR model *in vitro* and remove the protective effect of DHI *in vitro.*

Next, up-regulated miR-30d-5p or down-regulated JAK1 were present in the DR model of DHI-treated ARPE-19 cells to explore the effects of miR-30d-5p and JAK1 on the DR model *in vitro*. First, transfection was verified by qPCR (Figure 6(a)). It was conveyed by plate cloning that the proliferation of ARPE-19 cells was apparently decreased after elevating miR-30d-5p or knocking out JAK1, and the apoptosis was reverse by flow cytometry. In addition, Transwell found that the migration ability of DHI to ARPE-19 cells was further enhanced by elevation of miR-30d-5p or suppression of JAK1. The same result was found for tube forming ability (Figure 6(b–e)).

(a) The transfection efficiency verified via qPCR; (b) cell proliferation detected by plate cloning; (c) apoptosis detected via Flow cytometry; (d) cell migration detected via Transwell assay; (e) the ability of cytological tubule formation tested via Tubule formation assay. The data were expressed as mean ± SEM, N = 3; ^ *P* < 0.05, versus in-NC; + *P* < 0.05, versus oe-NC.

## 4. Discussion

DN and DR are common complications of DM. DN is a widespread and severe microvascular complication of DM [29]. As elevated diabetic patients, DN has become the main reason causing end-stage renal disease [30]. Besides, DR is also a microvascular complication of diabetes and is the main reason of vision loss in the general population worldwide. It is a retinal vascular abnormality caused by proliferative retinopathy (proliferative DR, PDR) or dysfunction and leakage of fluids and lipids into the retina [31]. DR and DN were mainly focused on in this study and the results were verified by *in vivo* experiment and *in vitro* cell model respectively.

DHI, derived from Salvia miltiorrhiza and Safflower, is widely applied in cardiovascular diseases and ischemic encephalopathy [32]. A study showed that DHI can repress the development of DR and DN in diabetic mice. On the one hand, db/db and db/m mice were purchased for DN. After injection of DHI, it was found that the blood glucose and body weight of the mice were clearly reduced, and the renal function and renal histopathology of the mice were improved, and the apoptosis was reduced. On the other hand, for DR, DR model was established in ARPE-19 cells treated with HG. After DHI treatment, the cell proliferation and migration abilities were apparently decreased, while apoptosis level was obviously elevated. In addition, some studies have shown that the characteristics of DR include retinal neovascularization and fibrous angiogenesis, resulting in retinal detachment [33]. DHI also effectively repressed the angiogenesis of ARPE-19 cells through tube formation experiments. These results all suggested that DHI has a certain therapeutic effect in DR and DN.

Many experiments have manifested that drug therapy may be momentous through modulating miRNAs. For example, sevoflurane suppresses the proliferation and invasion of neuroblastoma cells and induces apoptosis through miR-144-3p/YAP1 axis [34], and dexmedetomidine targets High-mobility group box 1 (HMGB1) protein through regulation of miR-205-5p, depressing cerebral ischemia/reperfusion inflammation and oxidative stress [35]. A certain association between DHI and miRNA has been documented. In addition, it has been reported that I/R injury can down-regulate miR-200a-3p [36], and miR-200-3p can depress the proliferation and reduce the apoptosis of DR cells. It was found that miR-200a-3p was obviously down-regulated in diabetic mice and high-glucose-induced DR model. MiR-200a-3p was elevated, so we speculated that DHI was highly likely to play a part through miR-200a-3p. In addition, we also found that inhibition of miR-200a-3p could reverse the therapeutic effect of DHI on db/db mice. However, in the DR cell model, the up-regulation of miR-200a-3p further reduced the proliferation, migration and tube formation ability of DHI-induced cells, and further elevated the level of apoptosis.

The Janus kinase signal converter and transcriptional activator (JAK-STAT) pathway is a newly discovered intracellular one that performs as interferon in the cell and involved not only in inflammatory response, but also in cell damage and apoptosis [37]. We first found that miR-200a-3p could target and regulate JAK1, and after inhibiting JAK1, it was found that JAK1 could further enhance the therapeutic effect of DHI on db/db mice and DR cells, suggesting that JAK1, as same as miR-200a-3p, was associated with the regulation of the development of DN and DR.

On the grounds of previous studies, the downstream mechanism of DHI’s therapeutic effect on DR and DN was further explored. It was proposed for the first time the mechanism of DHI’s targeted regulation of JAK1 via elevated miR-30d-5p. In the meantime, the function of miR-200a-3p/JAK1 axis on DR and DN was explored originally, which is a more reliable and powerful finding, providing more information for formulating intervention mechanisms of the prevention and treatment of early DN/DR. Of course, it has been documented that activated JAKs subsequently phosphorylates and activates signal transduction and transcriptional activator (STATs) [38]. The downstream mechanism of JAK1 in future studies will be further explored for further research, and its clinical impacts will also be explored in future studies.

## 5. Conclusion

In brief, this study finds that DHI represses DR and DN advancement, and the mechanism is possibly achieved via elevated miR-30d-5p and targeting JAK1, offering a favorable basis for DHI therapy of DR and DN, and a new understanding and potentially useful target.
